# Transition Metal Ion Complexes of Schiff-bases. Synthesis, Characterization and Antibacterial Properties

**DOI:** 10.1155/MBD.2001.137

**Published:** 2001

**Authors:** Zahid H. Chohan, Asifa Munawar, Claudiu T. Supuran

**Affiliations:** 1 Department of Chemistry Islamia University Bahawalpur Pakistan; 2 Universita degli Studi Dipartimento di Chimica Laboratorio di Chimica Inorganica e Bioinorganica Via Gino Capponi 7 Florence 50121 Italy

## Abstract

Some novel transition metal [Co(II), Cu(II), Ni(II) and Zn(II)] complexes of substituted pyridine Schiff-bases have been prepared and characterized by physical, spectral and analytical data. The synthesized Schiff-bases act as deprotonated tridentate for the complexation reaction with Co(II), Ni(II) and Zn(II) ions. The new compounds, possessing the general formula [M(L)_2_] where [M=Co(II), Cu(II), Ni(II) and Zn(II) and HL=HL^1^, HL^2^, HL^3^ and HL^4^] show an octahedral geometry. In order to evaluate the effect of metal ions upon chelation, the Schiff bases and their complexes have been screened for antibacterial activity against the strains such as *Escherichia coli*,*Staphylococcus aureus*, and *Pseudomonas aeruginosa*. The complexed Schiff bases have shown to be more antibacterial against one more bacterial species as compared to uncomplexed Schiff-bases.


### TRANSITION METAL ION COMPLEXES OF SCHIFF-BASES. SYNTHESIS, CHARACTERIZATION AND ANTIBACTERIAL PROPERTIES

Zahid H. Chohan*[1], Asifa Munawar[1] and Claudiu T. Supuran[2]

[1]Department of Chemistry, Islamia University, Bahawalpur, Pakistan
[2]Universita degli Studi, Dipartimento di Chimica, Laboratorio di Chimica Inorganica e Bioinorganica, Via Gino Capponi 7, 50121 Florence, Italy

### ABSTRACT

Some novel transition metal [Co(II), Cu(II), Ni(II) and Zn(II)] complexes of substituted pyridine Schiff-bases have been prepared and characterized by physical, spectral and analytical data. The synthesized Schiff-bases act as deprotonated tridentate for the complexation reaction with Co(II), Ni(II) and Zn(II) ions. The new compounds, possessing the general formula $[M(L)_2]$ where [M=Co(II), Cu(II), Ni(II) and Zn(II) and $HL=HL^1$, $HL^2$, $HL^3$ and $HL^4$] show an octahedral geometry. In order to evaluate the effect of metal ions upon chelation, the Schiff bases and their complexes have been screened for antibacterial activity against the strains such as *Escherichia coli*, *Staphylococcus aureus*, and *Pseudomonas aeruginosa*. The complexed Schiff bases have shown to be more antibacterial against one more bacterial species as compared to uncomplexed Schiff-bases.

### INTRODUCTION

Much attention[1-8] has been devoted by bioinorganic as well as by medicinal chemists to the relationship between the metal ions and their complexes as antitumour[9-12] and antibacterial[13-15] agents. *In vivo* studies have indicated that some biologically active compounds may become more carcinostatic and bacteriostatic upon chelation[16-22]. Such interactions of transition metal ions with amino acids, peptides and nucleic acids, are of immense biological importance[23-25]. Several reviews[26-29] showed that the metallo-organic chemistry of such compounds greatly influence their biological action highlighting the catalytic function metals[30-33] in many biological processes[34-37].

Several studies have revealed[38,39] that by condensation of salicylaldehyde with different heterocyclic compounds, derivatives with potent antibacterial and antifungal activity are obtained. Osman *et al*[40] prepared thiadiazole derived compounds of salicylaldehyde which were found to be highly potent antibacterial against *Bacillus cereus* and antifungal, against *Aspergillus niger*. Several compounds[41] incorporating piperazinyl guanidine, when condensed with salicylaldehyde were found to exhibit cardiovascular and vasodepressive activity. Studies of Shah *et al*[42] also showed thiazolidinone-derived salicylaldehydes to possess good antimicrobial activity. Keeping in view the significance of metals in biology, we have previously reported[43-47] several series of biologically active compounds and have evaluated the role of metal ions on their biological activity. In continuation to the same research topic, we report here some novel substituted pyridine Schiff bases obtained from salicylaldehyde. We have also studied the effect of substituents as well as metals ions on the biological activity of these derivatives.

### EXPERIMENTAL

### Material and Methods

All chemicals and solvents used were of Analar grade. All metal(II) salts were used as chlorides. IR spectra were recorded on a Philips Analytical PU 9800 FTIR spectrophotometer. UV-Visible spectra were obtained in DMF on a Hitachi U-2000 double-beam spectrophotometer. C, H and N analyses was carried out by Butterworth Laboratories Ltd. Conductance of the metal complexes was determined in DMF on a Hitachi YSI-32 model conductometer. Magnetic measurements were made on solid complexes using the Gouy method. Melting points were recorded on a Gallenkamp apparatus and are uncorrected.

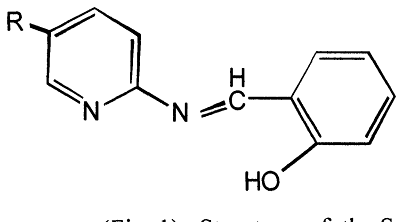

$HL^1$: R=OH
$HL^2$: $R=NO_2$
$HL^3$: $R=OCH_3$
$HL^4$: R=Br

(Fig. 1) Structure of the Schiff bases

**Preparation of Schiff base ($HL^1$)**

Salicylaldehyde (1.2 g, 1.1 mL, 0.01 M) in ethanol (10 mL) was added to an ethanol solution (20 mL) of 2-amino-5-hydroxypyridine (1.0 g, 0.01 M). Then 2-3 drops of conc.$H_2SO_4$ were added and the mixture refluxed for 2 h. On cooling, a solid product was formed which was filtered, washed with ethanol, then with ether and dried. Crystallization from hot ethanol gave $HL^1$. The same method was applied for the preparation of $HL^2$, $HL^3$ and $HL^4$ by using the corresponding reagents in the same molar ratio.

**Preparation of cobalt(II) complex of $HL^1$**

A warm ethanol solution (20 mL) of $HL^1$ (0.4 g, 0.002 M) was added to a magnetically stirred solution of cobalt chloride hexahydrate (0.24 g, 0.001 M) in distilled water (25 mL). The mixture was refluxed for 1 h and cooled to room temperature. On cooling, pink precipitates were formed which were filtered, washed with ethanol, acetone and ether, and dried by suction. Crystallization from aqueous ethanol (30:70) gave the desired metal complex (1). All other metal complexes were prepared respectively following the same method.

**Antibacterial studies**

The synthesized metal complexes, in comparison to the uncomplexed Schiff-bases were screened for their antibacterial activity against pathogenic bacterial strains, *Escherichia coli, Staphylococcus aureus* and *Pseudomonas aeruginosa*. The paper disc diffusion method[48,49] reported elsewhere was adopted for the determination of antibacterial activity.

### RESULTS AND DISCUSSION

**Physical properties**

The Schiff bases ($HL^1$-$HL^4$) (Fig. 1) were prepared by refluxing an appropriate amount of 5-hydroxy-, nitro-, methoxy- or bromo-substituted 2-aminopyridine and salicylaldehyde in hot ethanol in 1:1 molar ratio respectively. The structures of these Schiff bases were established with the help of their IR, NMR, and microanalytical data (Tables 1 and 2)

These Schiff bases were then used for the complexation with Co(II), Cu(II), Ni(II) and Zn(II) ions. All of the synthesized metal complexes [(1)-(16)] (Table 3) were air and moisture stable. These were prepared by the stoichiometric reaction of the corresponding metal salts (as chlorides) and the Schiff base in molar ratios M:L of 1:2. The complexes are intensely colored, amorphous solids, which decompose above 200° C. They are insoluble in common organic solvents such as ethanol, methanol, chloroform or acetone, but soluble in DMSO and DMF. Molar conductance values of the soluble complexes in DMF showed low values (12-19 $ohm^{-1}cm^2mol^{-1}$) indicating[50] them to be non-electrolytic.

Table I. Physical, spectral and analytical data of the Schiff bases

| Schiff base | IR ($cm^{-1}$) | Calc (Found) % C H N | M.P (°C) | Yield (%) |
|---|---|---|---|---|
| $HL^1$ $C_{12}H_{10}N_2O_2$ [214.0] | 3425 (br, Ph-OH), 3432 (br, Py-OH), 1630 (s, HC=N), 1620 (s, C=N). | 67.3 4.7 13.1 (67.8) (4.5) (13.0) | 118 | 78 |
| $HL^2$ $C_{12}H_9N_3O_3$ [243.0] | 3430 (br, OH), 1635 (s, HC=N), 1620 (s, C=N). | 59.3 3.7 17.3 (59.5) (3.9) (17.5) | 126 | 70 |
| $HL^3$ $C_{13}H_{12}N_2O_2$ [228.0] | 3430 (br, OH), 1635 (s, HC=N), 1625 (s, C=N). | 68.4 5.2 12.3 (68.3) (5.5) (12.0) | 137 | 75 |
| $HL^4$ $C_{12}H_8BrN_2O$ [275.9] | 3430 (br, OH), 1635 (s, HC=N), 1625 (s, C=N). | 52.2 2.9 10.1 (52.6) (3.2) (10.3) | 148 | 74 |

s=sharp, br=broad

**Infrared spectra**

IR spectra of the Schiff bases showed the absence of bands at 1735 and 3420 $cm^{-1}$ due to carbonyl ν(C=O) and ν($NH_2$) stretching vibrations and, instead, appearance of a strong new band at ~1635 $cm^{-1}$ assigned[51] to the azomethine, ν(HC=N) linkage. It suggested that amino and aldehyde moieties of the starting reagents are absent and have been converted into the azomethine moiety (Fig.1). The comparison of the IR spectra of the Schiff bases and their metal chelates (Table 4) indicated that the Schiff bases were principally coordinated to the metal atom in three ways, representing thus the ligands acting in a tridentate manner.

Table 2. $^1$H and $^{13}$C NMR data of the Schiff bases

| Schiff base | $^1$H NMR (DMSO-$d_6$) ppm | $^{13}$C NMR (DMSO-$d_6$) ppm |
|---|---|---|
| $HL^1$ | 7.3 (s, 1H, CH=N), 6.8 (m, 1H, Ph), 7.3 (m, 1H, Ph), 7.4 (m, 1H, Ph), 7.6 (m, 1H, Py), 7.7 (m, 1H, Ph), 7.9 (m, 1H, Py), 8.4 (m, 1H, Py), 9.9 (s, 1H, Ph-OH), 10.2 (s, 1H, OH, Py-OH). | 115.5, 126.5, 127.2, 130.3, 148.6, 155.1 (Ph), 150.7 (C=N),140.2, 149.3, 149.8, 155.2, 160.5 (Py). |
| $HL^2$ | 7.3 (s, 1H, CH=N), 7.5 (m, 1H, Ph), 7.6 (m, 1H, Ph), 7.7 (m, 1H, Py), 7.8 (m, IH, Ph), 7.9 (m, 1H, Ph), 8.1 (m, 1H, Py), 8.4 (m, 1H, Py), 10.1 (s, 1H, Ph-OH). | 115.4, 126.6, 127.1, 130.5, 148.5, 155.2 (Ph), 150.8 (C=N),140.3, 149.5, 149.8, 155.3, 160.7 (Py). |
| $HL^3$ | 3.6 (s, 3H, $OCH_3$, 7.3 (s, 1H, CH=N), 7.4 (m, 1H, Ph), 7.6 (m, 1H, Ph), 7.8 (m, 1H, Ph), 7. 9 (m, 1H, Py), 8.3 (m, 1H, Py), 8.4 (m, 1H, Py), 10.2 (s, 1H, Ph-OH). | 22.4 ($OCH_3$), 115.5, 126.7, 127.5, 130.2, 148.4, 155.1 (Ph), 150.6 (C=N), 140.2, 149.6, 149.7, 155.3, 160.6 (Py). |
| $HL^4$ | 7.3 (s, 1H, CH=N), 7.5 (m, 1H, Ph), 7.7 (m, 1H, Ph), 7.8 (m, 1H, Ph), 7.9 (m, 1H, Ph), 8.1 (m, 1H, Py), 8.3 (m, 1H, Py), 8. 4 (m, 1H, Py), 10.2 (s, 1H, Ph-OH). | 115.6, 126.6, 127.2, 130.5, 148.6, 155.3 (Ph), 150.8 (C=N),140.4, 149.7, 149.8, 155.4, 160.7 (Py). |
| 13 | 7.3 (s, 1H, CH=N), 6.9 (m, 1H, Ph), 7.4 (m, 1H, Ph), 7.5 (m, 1H, Ph), 7.6 (m, 1H, Py), 7.8 (m, 1H, Ph), 8.1 (m, 1H, Py), 8.4 (m, 1H, Py), 10.2 (s, 1H, OH, Py-OH). | 115.6, 126.5, 127.3, 130.5, 148.6, 155.2 (Ph), 150.9 (C=N),140.4, 149.5, 149.8, 155.3, 160.5 (Py). |
| 14 | 7.4 (s, 1H, CH=N), 7.6 (m, 1H, Ph), 7.7 (m, 1H, Ph), 7.8 (m, 1H, Py), 7.9 (m, IH, Ph), 8.0 (m, 1H, Ph), 8.1 (m, 1H, Py), 8.4 (m, 1H, Py). | 115.5, 126.7, 127.2, 130.5, 148.6, 155.3 (Ph), 150.9 (C=N),140.5, 149.5, 149.8, 155.4, 160.7 (Py). |
| 15 | 3.7 (s, 3H, $OCH_3$, 7.4 (s, 1H, CH=N), 7.5 (m, 1H, Ph), 7.7 (m, 1H, Ph), 7.8 (m, 1H, Ph), 8.1 (m, 1H, Py), 8.3 (m, 1H, Py), 8.6 (m, 1H, Py). | 22.6 ($OCH_3$), 115.6, 126.7, 127.7, 130.4, 148.4, 155.3 (Ph), 150.8 (C=N), 140.3, 149.6, 149.8, 155.4, 160.7 (Py). |
| 16 | 7.5 (s, 1H, CH=N), 7.6 (m, 1H, Ph), 7.8 (m, 1H, Ph), 7.9 (m, 1H, Ph), 8.1 (m, 1H, Ph), 8.2 (m, 1H, Py), 8.3 (m, 1H, Py), 8. 5 (m, 1H, Py). | 115.7, 126.6, 127.5, 130.3, 148.7, 155.4 (Ph), 150.9 (C=N),140.4, 149.5,149.7, 155.5, 160.7 (Py). |

a) The band appearing at 1635 $cm^{-1}$ due to the azomethine was shifted to lower frequency by ~10-15 $cm^{-1}$ Indicating[52] participation of the azomethine nitrogen in the complexation.

b) The band at 1620 assigned to pyridine ring ν(C=N) nitrogen also shifted to lower frequency by ~15-25 $cm^{-1}$ which was indicative of the involvement of ring nitrogen of pyridine in chelation.

c) A band appearing at 3425 $cm^{-1}$ assigned to ν(OH) in the Schiff base compounds was not found in the spectra of their metal complexes indicating deprotonation and coordination of the hydroxyl oxygen to the metal atom.

d) Further conclusive evidence of the coordination of these Schiff base compounds with the metals, was shown by the appearance of weak low frequency new bands at 525-530 and 455-460 $cm^1$. These were assigned[53] to the metal-nitrogen ν(M-N) and metal-oxygen ν(M-O) respectively. These new bands were observable only in the spectra of the metal complexes and not in the spectra of its uncomplexed Schiff base compounds thus confirming participation of these hetero groups (O or N) in the coordination.

**NMR spectra**

The $^1$H NMR spectra of the Schiff bases and of their Zn(II) complexes taken in DMSO-$d_6$ are listed in Table 2. The Schiff bases exhibited signals due to all the expected protons in their expected region and have been identified from the integration curve found to be equivalent to the total number of protons deduced from their proposed structures. These were compared with the reported[54] signals of the known identical compounds and give further support for the compositions of the new ligands as well as their complexes suggested by their IR and elemental analyses data. Comparison of the chemical shifts of the uncomplexed Schiff bases with those of the corresponding complex show that some of the resonance signals experience shifts upon the complexation. In each case, the protons assigned due to heteroaromatic (HC=N), azomethine (HC=N), hydroxyl group (OH) and substituted aromatic were found at around ~ 8.8, 7.3, 9.9 and 6.8-7.7 ppm in the spectra of the ligands. The protons due to heteroaromatic, azomethine and substituted aromatic undergo shift towards downfield by 0.9-1.0 ppm in the complexes indicating coordination of these groups with the metal atom. Also, protons due to hydroxyl group (OH) were found absent in the spectra of the complexes. The absence of these signals suggested[55] the deprotontion of the hydroxyl group and the involvement of the oxygen atom in complexation.

Table 3. Physical and analytical data of the metal(II) chelates

| No | Metal chelate/ Mol. Formula | Yield (%) | M.P ($^{o}C$) (decomp) | B.M. ($\mu_{eff}$) | Calc (Found)% C H N |
|---|---|---|---|---|---|
| 1 | $[Co(L^1)_2]$ [484.9] $C_{24}H_{18}CoN_4O_4$ | 60 | 218-220 | 4.2 | 59.4 3.7 11.5 (59.1) (3.3) (11.9) |
| 2 | $[Co(L^2)_2]$ [542.9] $C_{24}H_{16}CoN_6O_6$ | 56 | 220-222 | 4.7 | 53.0 .2.9 15.5 (53.4) (2.4) (15.4) |
| 3 | $[Co(L^3)_2]$ [512.9] $C_{26}H_{22}CoN_4O_4$ | 58 | 221-223 | 4.5 | 60.8 4.3 10.9 (60.7) (4.1) (10.5) |
| 4 | $[Co(L^4)_2]$ [584.7] $C_{22}H_{14}CoBr_2N_4O_2$ | 61 | 225-227 | 4.3 | 45.2 2.4 9.6 (45.6) (2.3) (9.8) |
| 5 | $[Cu(L^1)_2]$ [489.5] $C_{24}H_{18}CuN_4O_4$ | 57 | 218-220 | 1.6 | 58.8 3.7 11.4 (58.7) (3.5) (11.2) |
| 6 | $[Cu(L^2)_2]$ [547.5] $C_{24}H_{16}CuN_6O_6$ | 58 | 228-230 | 1.4 | 52.6 2.9 15.3 (52.5) (2.5) (15.4) |
| 7 | $[Cu(L^3)_2]$ [517.5] $C_{26}H_{22}CuN_4O_4$ | 58 | 212-214 | 1.5 | 60.3 4.3 10.8 (60.5) (4.5) (10.4) |
| 8 | $[Cu(L^4)_2]$ [589.3] $C_{22}H_{14}CuBr_2N_4O_2$ | 60 | 226-228 | 1.4 | 44.8 2.4 9.5 (45.1) (2.2) (9.3) |
| 9 | $[Ni(L^1)_2]$ [484.7] $C_{24}H_{18}NiN_4O_4$ | 61 | 220-222 | 3.3 | 59.4 3.7 11.5 (59.5) (3.4) (11.3) |
| 10 | $[Ni(L^2)_2]$ [542.7] $C_{24}H_{16}NiN_6O_6$ | 56 | 225-228 | 3.1 | 53.1 2.9 15.5 (53.4) (2.7) (15.4) |
| 11 | $[Ni(L^3)_2]$ [512.7] $C_{26}H_{22}NiN_4O_4$ | 58 | 228-230 | 3.4 | 60.9 4.3 11.0 (60.7) (4.5) (11.3) |
| 12 | $[Ni(L^4)_2]$ [584.5] $C_{22}H_{14}NiBr_2N_4O_2$ | 60 | 222-224 | 3.2 | 45.2 2.4 9.6 (45.5) (2.3) (9.8) |
| 13 | $[Zn(L^1)_2]$ [491.4] $C_{24}H_{18}ZnN_4O_4$ | 60 | 215-217 | Dia | 58.6 3.7 11.4 (58.9) (3.4) (11.3) |
| 14 | $[Zn(L^2)_2]$ [549.4] $C_{24}H_{16}ZnN_6O_6$ | 58 | 218-220 | Dia | 52.4 2.9 15.3 (52.7) (2.5) (15.1) |
| 15 | $[Zn(L^3)_2]$ [519.4] $C_{26}H_{22}ZnN_4O_4$ | 61 | 222-224 | Dia | 60.1 4.2 10.8 (60.2) (4.3) (10.5) |
| 16 | $[Zn(L^4)_2]$ [591.2] $C_{22}H_{14}ZnBr_2N_4O_2$ | 62 | 225-227 | Dia | 44.7 2.4 9.5 (44.5) (2.3) (9.7) |

**Magnetic moments and UV-visible spectra**

The room temperature magnetic moment of the solid cobalt (II) complexes was found to lie in the range (4.2-4.7 B.M), indicative[52] of three unpaired electrons per Co (II) ion in an octahedral environment. The Cu (II) complexes showed $\mu_{Fe}$ values in the range (1.4-1.6 B.M) indicative of one unpaired electron per Cu (II) ion suggesting[56] these complexes within the range consistent to spin-free distorted octahedral geometry. Similarly the In (II) complexes showed $\mu_{Fe}$ values in the range (3.1-3.4 B.M), corresponding[57] to two unpaired electrons per Zn(II) ion for their ideal six-coordinated configuration. The Zn(II) complexes were all found diamagnetic.

The electronic spectra of the Co(II) chelates showed three bands observed at 8780-8815, 17560-18425 and 30210-30575 $cm^{-1}$ which may be assigned to ${}^4T_{1g} \rightarrow {}^4T_{2g}(F)$, ${}^4T_{1g} \rightarrow {}^4A_{2g}(F)$ and ${}^4T_{1g} \rightarrow {}^4T_{1g}(P)$ transitions respectively and are suggestive[55859] of the octahedral geometry around the cobalt ions.

The Cu(II) complexes showed three absorption bands between 10 Dq band for a distorted octahedral geometry corresponding[60,61] to the transitions $2E_g \rightarrow 2T_{2g}$. The bands at 22152-22355 and 30550-30645 $cm^{-1}$ may be due to intra-ligand charge transfer transitions.

The Ni(II) complexes exhibited three spin-allowed bands at 9815-10145, 15945-16250 and 28540-29210 $cm^{-1}$ assignable[62,63] respectively, to the transitions $^3A_{2g}(F) \rightarrow {}^3T_{2g}(F)(\nu_1)$, $^3A_{2g}(F) \rightarrow {}^3T_{1g}(F)(\nu_2)$ and $^3A_{2g}(F) \rightarrow {}^3T_{2g}(P)(\nu_3)$ which were characteristic of their octahedral geometry (Fig 2).

Table 4. IR and UV-visible spectral data of the metal(II) chelates

| No | IR ($cm^{-1}$) | $\lambda_{max}$ ($cm^{-1}$) |
|---|---|---|
| 1 | 1625 (s, HC=N), 1580 (s, C=N), 525 (ms, M-N), 455 (ms, M-O) | 30210, 18425, 8815 |
| 2 | 1620 (s, HC=N), 1585 (s, C=N), 525 (ms, M-N), 455 (ms, M-O) | 30575, 17850, 8795 |
| 3 | 1620 (s, HC=N), 1590 (s, C=N), 530 (ms, M-N), 460 (ms, M-O) | 30545, 17950, 8810 |
| 4 | 1620 (s, HC=N), 1590 (s, C = N), 530 (ms, M-N), 460 (ms, M-O) | 30550, 17560, 8780 |
| 5 | 1625 (s, HC=N), 1585 (s, C = N), 525 (ms, M-N), 460 (ms, M-O) | 30565, 22255 |
| 6 | 1620 (s, HC=N), 1580 (s, C=N), 530 (ms, M-N), 455 (ms, M-O) | 30575, 22350 |
| 7 | 1625 (s, HC=N), 1585 (s, C=N), 525 (ms, M-N), 460 (ms, M-O) | 30645, 22265 |
| 8 | 1625 (s, HC=N), 1585 (s, C=N), 530 (ms, M-N), 455 (ms, M-O) | 30550, 22180 |
| 9 | 1620 (s, HC=N), 1580 (s, C=N), 525 (ms, M-N), 455 (ms, M-O) | 28540, 16275, 9815 |
| 10 | 1620 (s, HC=N), 1585 (s, C=N), 525 (ms, M-N), 460 (ms, M-O) | 29210, 15945, 10145 |
| 11 | 1625 (s, HC=N), 1590 (s, C=N), 530 (ms, M-N), 460 (ms, M-O) | 28875, 16250, 9980 |
| 12 | 1620 (s, HC=N), 1585 (s, C=N), 525 (ms, M-N), 455 (ms, M-O) | 28910, 16155, 9875 |
| 13 | 1625 (s, HC=N), 1590 (s, C=N), 530 (ms, M-N), 455 (ms, M-O) | 28450 |
| 14 | 1625 (s, HC=N), 1595 (s, C=N), 530 (ms, M-N), 460 (ms, M-O) | 28510 |
| 15 | 1620 (s, HC=N), 1585 (s, C=N), 525 (ms, M-N), 460 (ms, M-O) | 28475 |
| 16 | 1625 (s, HC=N), 1585 (s, C=N), 525 (ms, M-N), 455 (ms, M-O) | 28500 |

s=sharp, ms=medium sharp

The diamagnetic zinc(II) complexes did not show any d-d bands and their spectra are dominated only by charge transfer bands. The charge transfer band at 28450-28510 $cm^{-1}$ was assigned[63,64] due to transition $^2E_g \rightarrow {}^2T_{2g}$ possibly in an octahedral environment.

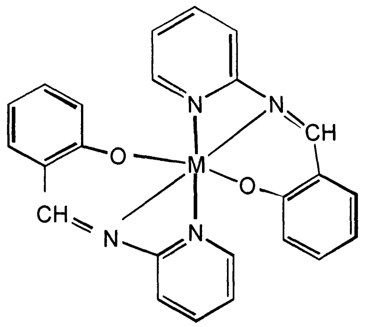

R=OH ($HL^1$)

R=$NO_2$ ($HL^2$)

R=$OCH_3$ ($HL^3$)

R=Br ($HL^4$)

M=Co(II), Cu(II), Ni(II) or Zn(II)

(Fig. 2) Proposed structure of the metal(II) complex

**Antibacterial properties**

The title Schiff bases and their metal chelates were evaluated for their antibacterial activity against the strains *Escherichia coli (a), Staphylococcus aureus (b) and Pseudomonas aeruginosa (c).* The compounds were tested at a concentration of 30 µg/0.01 mL in DMF solution using the paper disc diffusion method. The susceptibility zones were measured in diameter (mm) and the results are reproduced in Table 5. The susceptibility zones measured were the clear zones around the discs killing the bacteria.

All the Schiff bases and their complexes individually exhibited varying degrees of inhibitory effects on the growth of the tested bacterial species. The antibacterial results evidently show that the activity of the Schiff base compounds became more pronounced when coordinated to the metal ions. All metal ions have varying antibacterial influence on bacterial species. The Co(II) complex of $HL_1$ was more antibacterial against one species and less against the other as compared to the Co(II) complex of the other Schiff bases. Same results were found for other metal complexes. It is however, not possible to make out exactly which metal ion is playing more antibacterial role against one or the other bacterial species but, it is definitive that metal ions do play a significant role in enhancing the antibacterial activity of antibacterial agents on chelation. It is suggested that in the chelated complex, the positive charge of the metal ion is partially shared with the donor atoms and there is π-electron delocalization over the whole chelate ring. This increases the lipophilic character of the metal chelate and favors its permeation through lipoid layers of the bacterial membranes. It is also suspected that factors such as solubility, dipole moment and cell permeability mechanisms are also influenced by the presence of the metal ions, which are responsible in enhancing this role of metals as bactericidal. Our *in vitro* studies are in progress, which would help us in determining further the actual mechanism involved in enhancing this activity.

Table 5. Antibacterial activity data of the Schiff bases and its metal(II) chelates

| Schiff Base/ Chelate | Microbial Species a | b | c |
|---|---|---|---|
| $HL^1$ | ++ | + | ++ |
| $HL^2$ | ++ | ++ | ++ |
| $HL^3$ | +++ | ++ | +++ |
| $HL^4$ | +++ | ++ | +++ |
| 1 | +++ | +++ | ++ |
| 2 | +++ | ++ | +++ |
| 3 | +++ | +++ | +++ |
| 4 | ++++ | +++ | +++ |
| 5 | +++ | +++ | +++ |
| 6 | ++++ | ++ | +++ |
| 7 | ++ | +++ | + |
| 8 | +++ | +++ | +++ |
| 9 | +++ | +++ | +++ |
| 10 | +++ | +++ | ++ |
| 11 | ++ | +++ | ++++ |
| 12 | +++ | +++ | +++ |
| 13 | ++ | +++ | ++++ |
| 14 | +++ | ++ | +++ |
| 15 | +++ | +++ | ++ |
| 16 | +++ | +++ | +++ |

a= *Escherichia coli*, b= *Staphylococcus aureus*, c= *Pseudomonas aeruginosa*
Inhibition zone diameter mm (% inhibition): +, 6-10 (27-45 %); ++, 10-14 (45-64 %); +++, 14-18 (64-82 %); ++++, 18-22 (82-100 %). Percent inhibition values are relative to inhibition zone (22 mm) of the most active compound with 100 % inhibition.

### ACKNOWLEDGEMENT
The authors gratefully acknowledge the Department of Pathology, Qaid-e-Azam Medical College, Bahawalpur, Pakistan, in undertaking the antibacterial studies.

### REFERENCES
1. A. Scozzafava, L. Menabuoni, F. Mincione, G. Mincione and C.T. Supuran, *Bioorg. Med. Chem. Lett*, **11**, 575 (2001);
2. A. Scozzafava and C. T. Supuran *J. Med. Chem*, **43**, 3677 (2000).
3. G. Alzuet, J. Casanova, J. Borras, S. Garcia-Granda, A. Gutierrez-Rodriguez and C. T. Supuran, *Inorganica Chimica Acta*, **273**, 334 (1998).
4. C.T. Supuran, A. Scozzafava *Eur. J. Med. Chem*, **35**, 867(2000).
5. D. R. Williams, *The Metals of Life*", Van Nostrand, London, (1971).
6. R. J. P. William, "*Quart. Rev*, **24**, 331 (1970).
7. R. J. P. William, "*Bioinorganic Chemistry*", American Chemical Society, Washington, (1971).
8. D. H. Brown, W. E. Smith, J. W. Teape and A. J. Lewis, *J. Med. Chem*, **23**, 729 (1980).
9. B. Rosenberg and L. V. Camp, *Cancer. Res*, **30**, 1799 (1970)
10. M. J. Clare and J. D. Heeschele, *Bioinorg. Chem*, **2**, 187 (1973).
11. V. L. Narayanan, M. Nasr and K. D. Paull, in "*Tin-Based Antitumor Drugs*", M. Gielen (Ed), NATO ASI Series, Vol. H37, Springer Verlag, Berlin (1990).
12. A. J. Crowe, in "*Metal Based Antitumor Drugs*", M. Gielen (Ed), Vol 1, Freund, London (1988).
13. M. J. Seven and L. A. Johnson, "*Metal Binding in Medicine*", Lippincott Co, Philadelphia, PA, 4th Ed (1960).
14. R. S. Srivastava, *Ind. J. Chem*, **29A**, 1024 (1990).
15. V. K. Patel, A. M. Vasanwola and C. R. Jejurkas, *Ind. J. Chem*, **28A**, 719 (1989).
16. A. Scozzafava and C. T. Supuran, *J. Med. Chem*, **43**, 3677 (2000).
17. C. T. Supuran, A. Scozzafava, L. Menabuoni, F. Mincione, F. Briganti and G. Mincione, *Metal-Based Drugs*, **6**, 67 (1999).

18. C. T. Supuran, F. Mincione, A. Scozzafava, F. Briganti, G. Mincione and M. A. Ilies, *Eur. J. Med. Chem,* **33**, 247 (1998).
19. C. T. Supuran, A. Scozzafava, I. Saramet, M. D. Banciu, *J. Enzyme. Inhib*, **13**, 177 (1998).
20. C. T. Supuran and A. Scozzafava, *J. Enzyme. Inhib*, **12**, 37 (1997).
21. G. Mincione, A. Scozzafava and C. T. Supuran, *Metal-Based Drugs*, **4**, 27 (1997).
22. A. Scozzafava and C. T. Supuran, *Metal-Based Drugs*, **4**, 19 (1997).
23. J. M. Pratt, *Inorganic Chemistry of Vitamin B*", Academic Press, London (1970).
24. S. K. Shapiro and F. Schlenk, "*Transmethylation and Methionine Biosynthesis*", University of Chicago Press, Chicago (1965).
25. J. M. Walshe, *Brit. J. Hospit. Med*, **6**, 45 (1970).
26. E. Ochial, *Coord. Chem. Rev*, **3**, 49 (1968).
27. F. Frieden, *Sci. Amer*, **218**, 102 (1968).
28. R. J. P. William, *Chem. Rev*, **1**, 13 (1968).
29. E. Sinn and C. M. Harris, *Coord. Chem. Rev*, **4**, 391 (1969).
30. M. Barboiu, C. T. Supuran, A. Scozzafava, C. Guran, P. Diaconescu, M. Bojin, V. Iluc and L. Cot, *Metal-Based Drugs*, **6**, 101 (1999).
31. D. de Vos, P. Clements, S. M. Pyke, D. R. Smyth and E. R. T. Tiekink, *Metal-Based Drugs*, **6**, 31 (1999).
32. M. Geraghly, M. McCann, M. Devereux, F. Cronin, M. Curran and V. McKee, *Metal-Based Drugs*, **6**, 41 (1999).
33. K. Sharma and R. V. Singh, *Metal-Based Drugs*, **7**, 1 (2000).
34. T. Pandey and R. V. Singh, *Metal-Based Drugs*, **7**, 7 (2000).
35. A. Mastrolorenzo and C. T. Supuran, *Metal-Based Drugs*, 7, 49 (2000).
36. A. Scozzafava, L. Menabuoni, F. Mincione, G. Mincione and C. T. Supuran, Bioorg. *Med. Chem. Lett*, **11**, 575 (2001).
37. C. Walsh, *Science*, **409**, 226 (2001).
38. R. V. Singh, *Synth. React. Inorg. Met-Org. Chem*, **16**, 21 (1986).
39. M. A. Mohammad, M. M. El-Enamy and E. L. Basies, *Egypt. J. Pharm. Sci*, **22**, 9 (981).
40. A. M. Osman, K. Hassan, M. E. Kashaf, M. A. Maghraby and M. A. Hassan, *J. Ind. Chem. Soc*, **56**, 521 (1979).
41. B. Dash and M. Patra, *Ind. J. Chem*, **19B**, 894 (1980).
42. V. H. Shah and A. R. Parkash, *Ind. J. Chem*, **54**, 41 (1982).
43. Z. H. Chohan and S. K. A. Sherazi, *Synth. React. Inorg. Met.-Org. Chem*, **29**, 105 (1999).
44. Z. H. Chohan and S. Kausar, *Metal-Based Drugs*, 7, 17 (2000).
45. Z. H. Chohan and M. Praveen, *Appl. Organometal. Chem*, **14**, 376 (2000).
46. Z. H. Chohan and M. Praveen, *Synth. React. Inorg. Met.-Org. Chem*, **30**, 175 (2000).
47. Z. H. Chohan and M. Praveen, *Appl. Organometal. Chem*, (In Press).
48. Z. H. Chohan, *Metal-Based Drugs*, **7**, 177 (2000).
49. Z. H. Chohan, M. A. Farooq and M. S. Iqbal, *Metal-Based Drugs*, **7**, 133 (2000).
50. W. J. Geary, *Coord. Chem. Rev*, **7**, 81 (1971).
51. L. J. Bellamy, "*The Infrared Spectra of Complex Molecules*", 3rd Ed, Methuen, London, (1966).
52. M. Yongxiang, Z. Zhengzhi, M. Yun and Z. Gang, *Inorg. Chim. Acta*, **165**, 185 (1989).
53. K. Nakamoto, "*Infrared Spectra of Inorganic and Coordination Compounds*", 2nd Ed, Wiley Interscience, New York, (1970).
54. D. H. Williams and I. Fleming, "*Spectroscopic Methods in Organic Chemistry*", 4th Ed, Mc Graw Hill, London, (1989).
55. Z. Hong-Yun, C. Dong-Li, C. Pei-Kun, C. De-Ji, C. Guang-Xia and Z. Hong-Quan, *Polyhedron*, **11**, 2313 (1992).
56. B. N. Figgis, "*Introduction to Ligand Fields*", J. Wiley, New York, (1976).
57. B. P. Lever, "*Inorganic Electronic Spectroscopy*", Elsevier, Amsterdam, (1984).
58. D. Liehr, *J. Phys. Chem*, **67**, 1314 (1967).
59. T. M. Dunn, J. Lewis and R. C. Wilkins, *The Visible and Ultraviolet Spectra of Complex Compounds in Modern Coordination Chemistry*", Interscience, New York, (1960).
60. A. B. P. Lever, J. Lewis and R. S. Nyholm, *J. Chem. Soc*, 4761 (1964).
61. R. L. Carlin,., "*Transition Metal Chemistry*", Ed, R. L. Carlin, Vol 1, Marcel Decker, New York, (1965).
62. D. W. Meek, R. S. Drago and T. S. Piper, *Inorg. Chem.*, **1**, 285 (1962).
63. R. S..Drago, "*Physical Methods in Inorganic Chemistry*", Reinhold, New York, (1965).
64. B. N. Figgis and J. Lewis, *Prog. Inorg. Chem.*, **6**, 87 (1964).

**Received: June 12, 2000 – Accepted: June 18, 2000 –**
**Accepted in publishable format: June 19, 2001**

